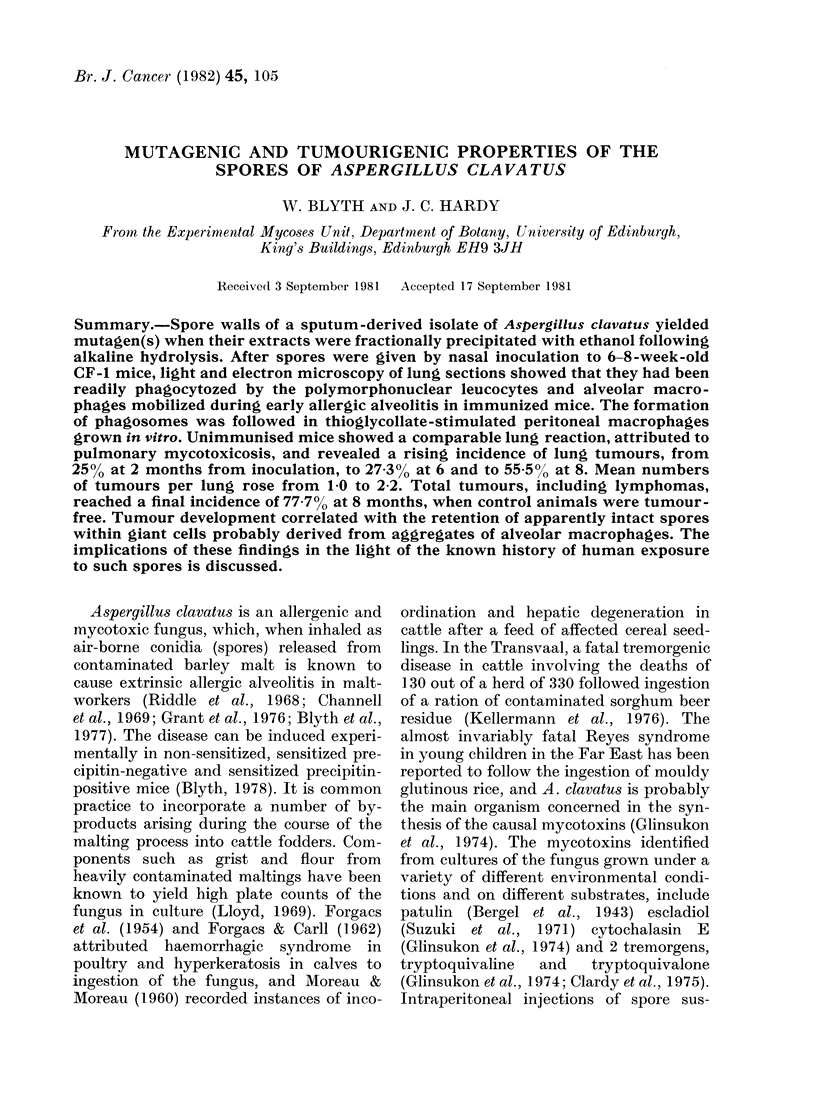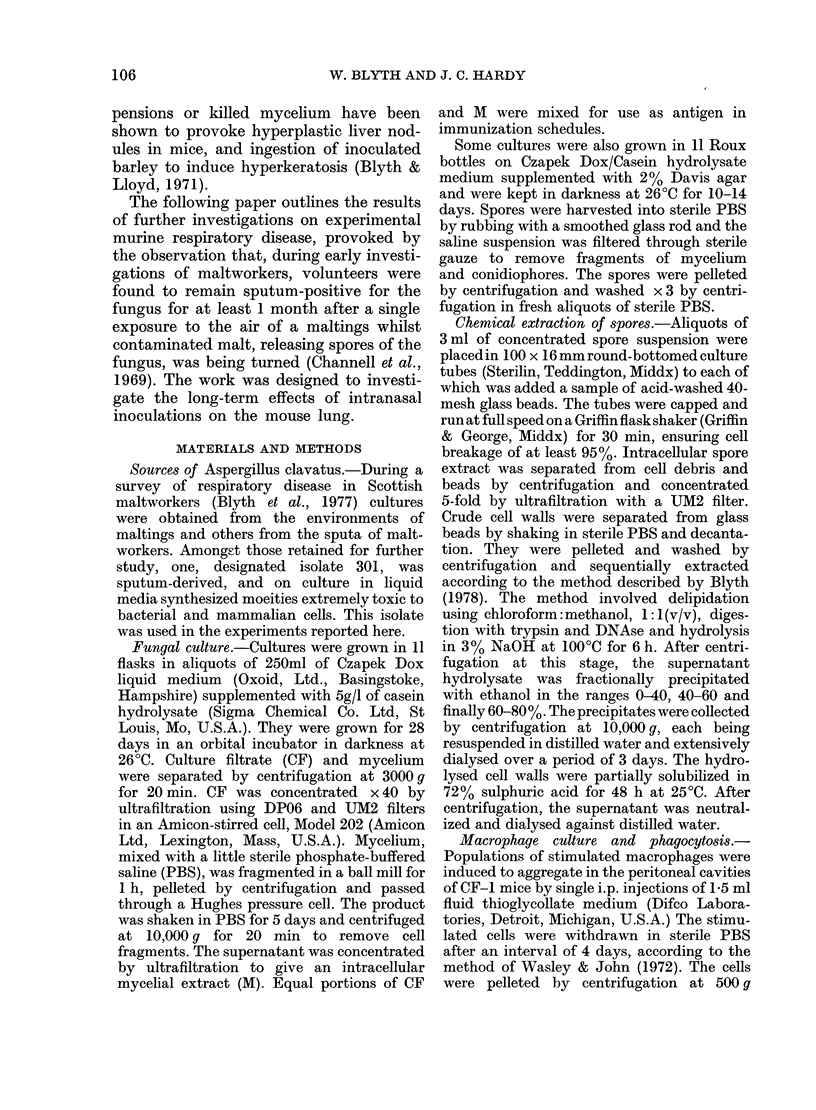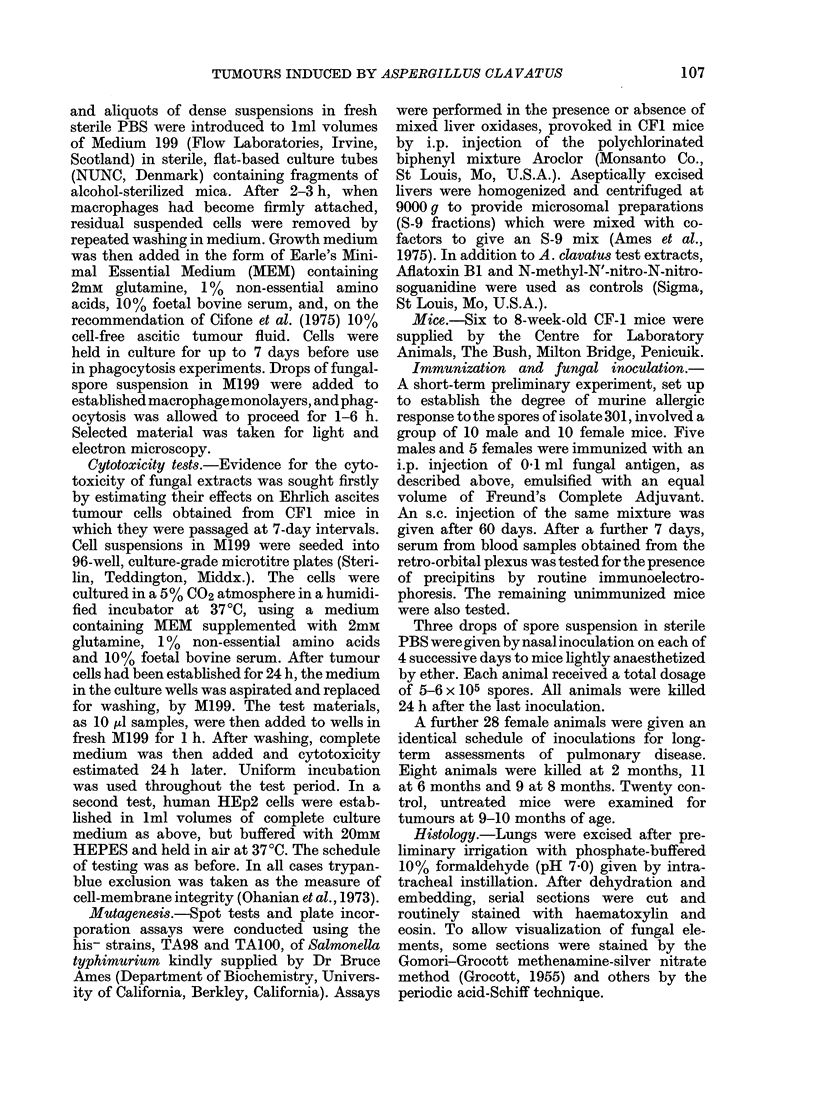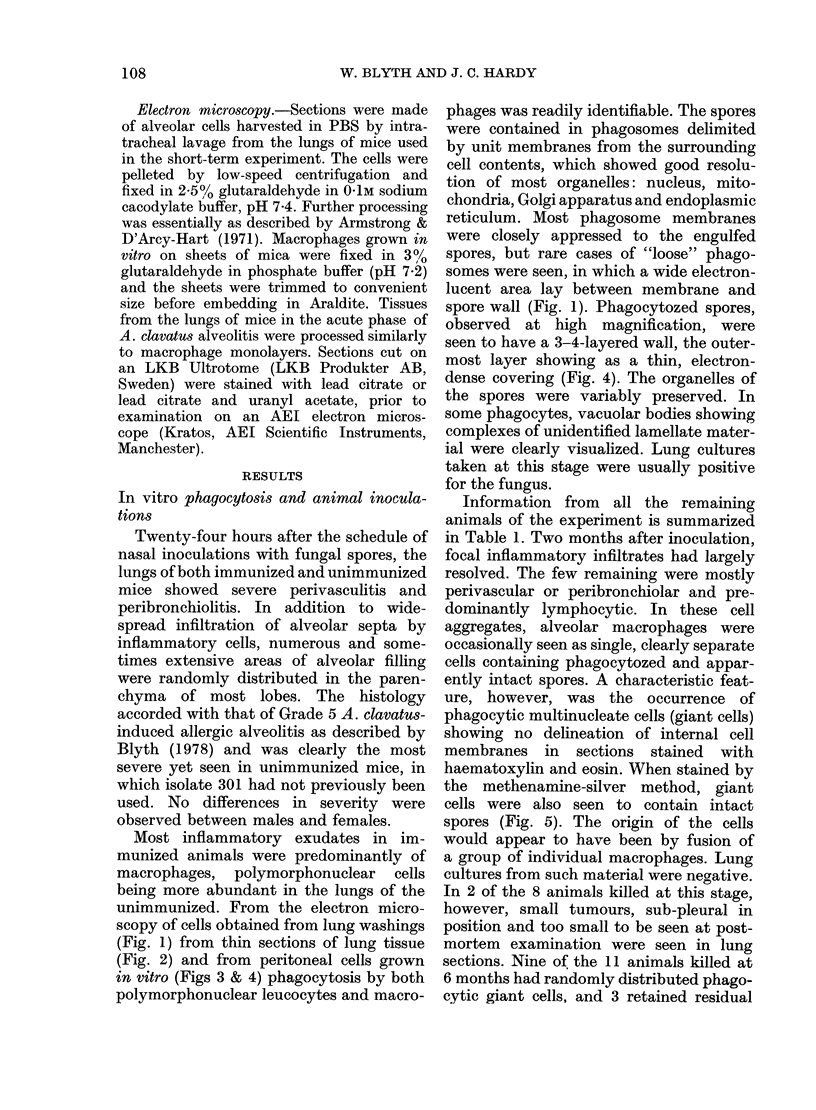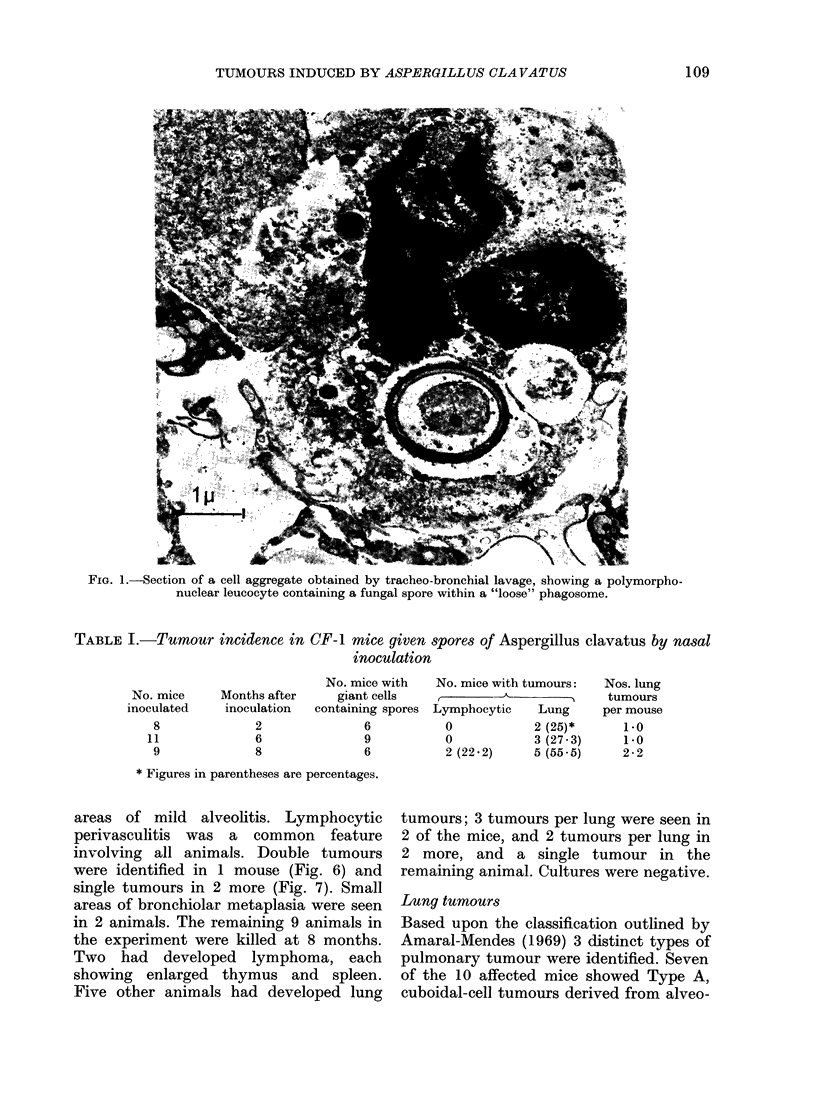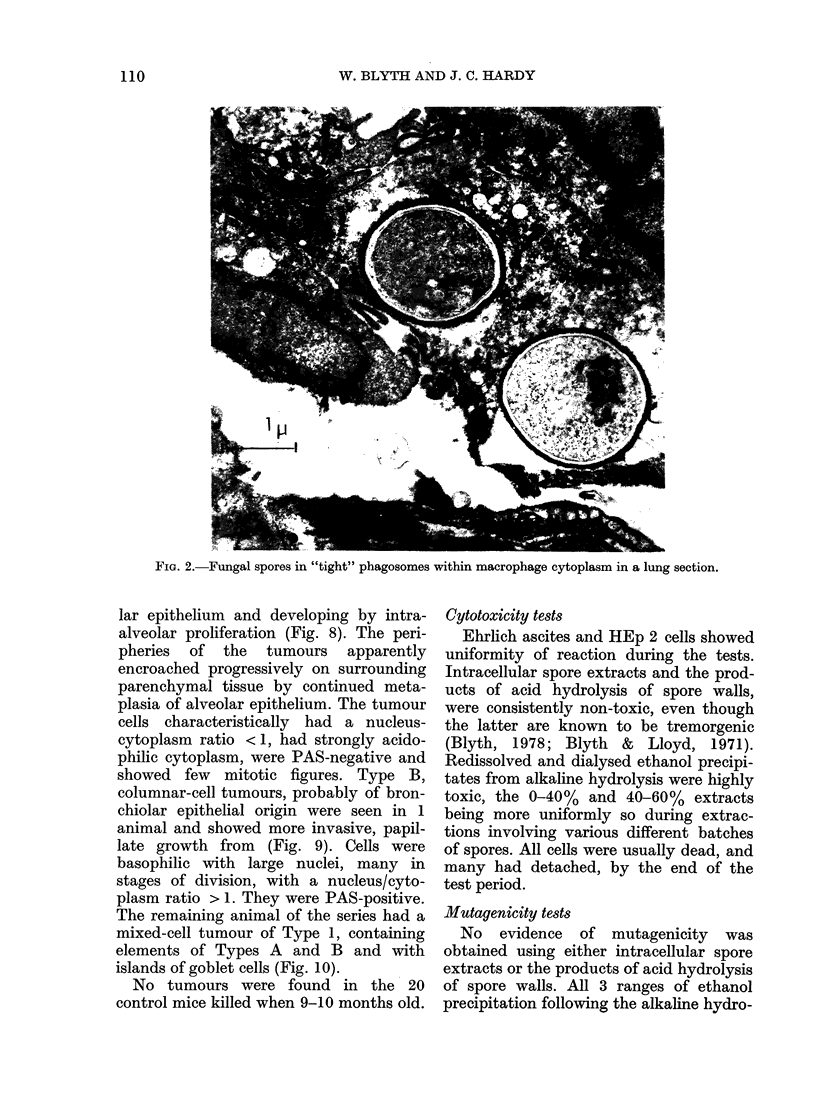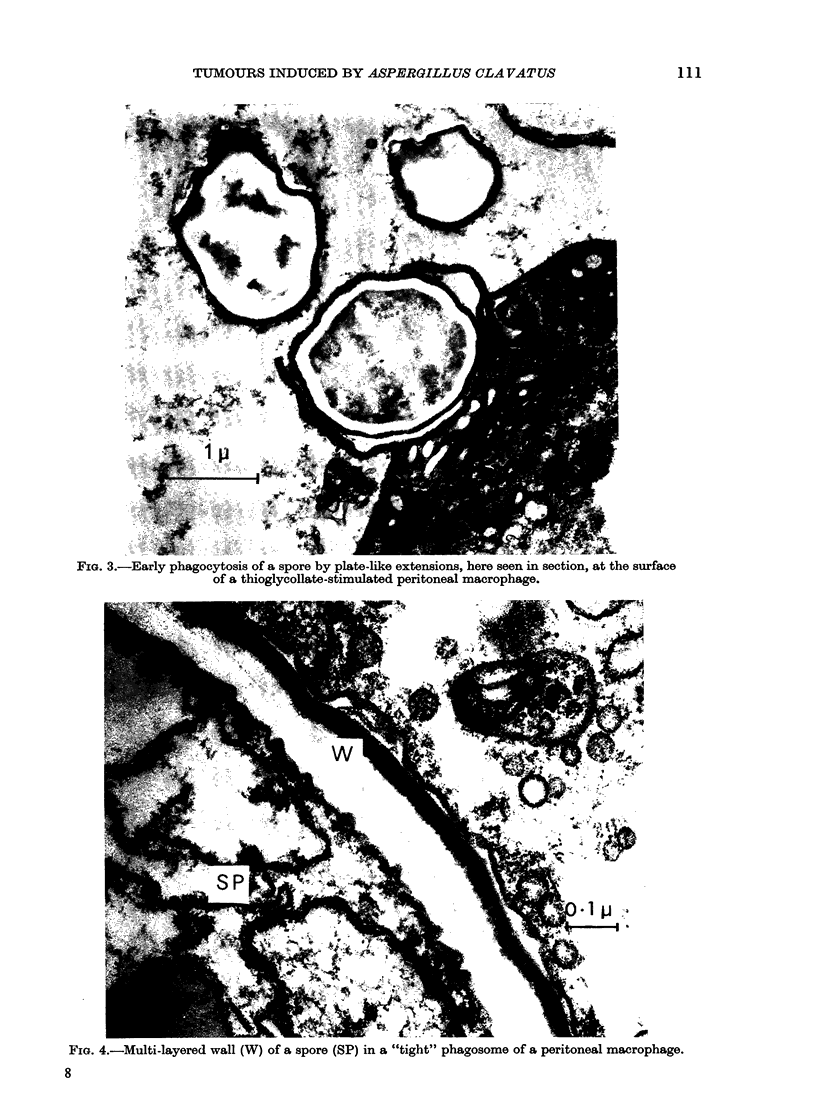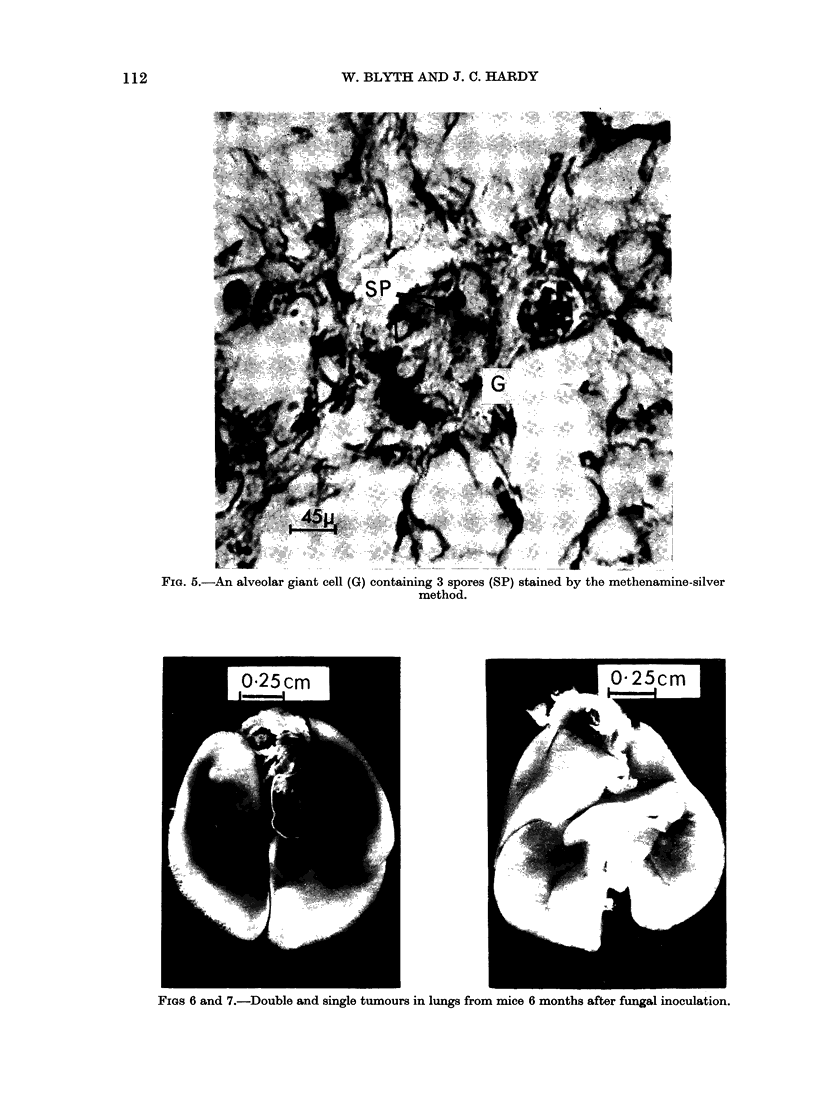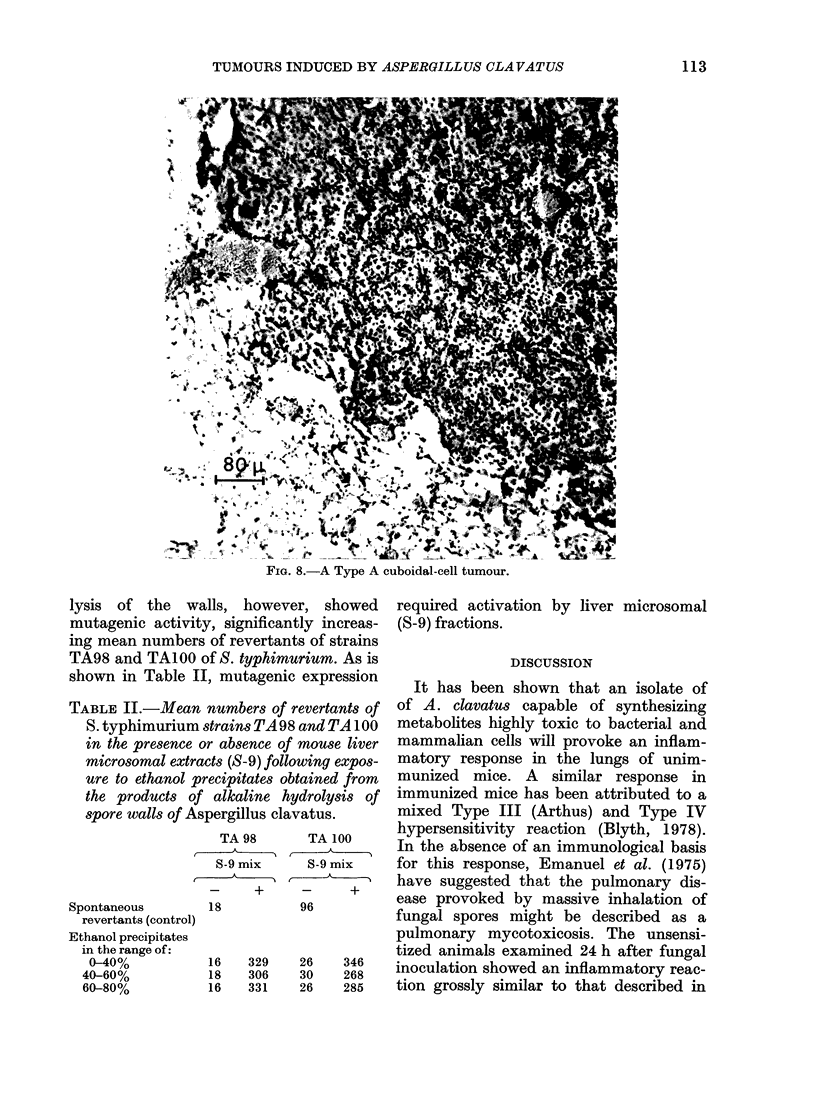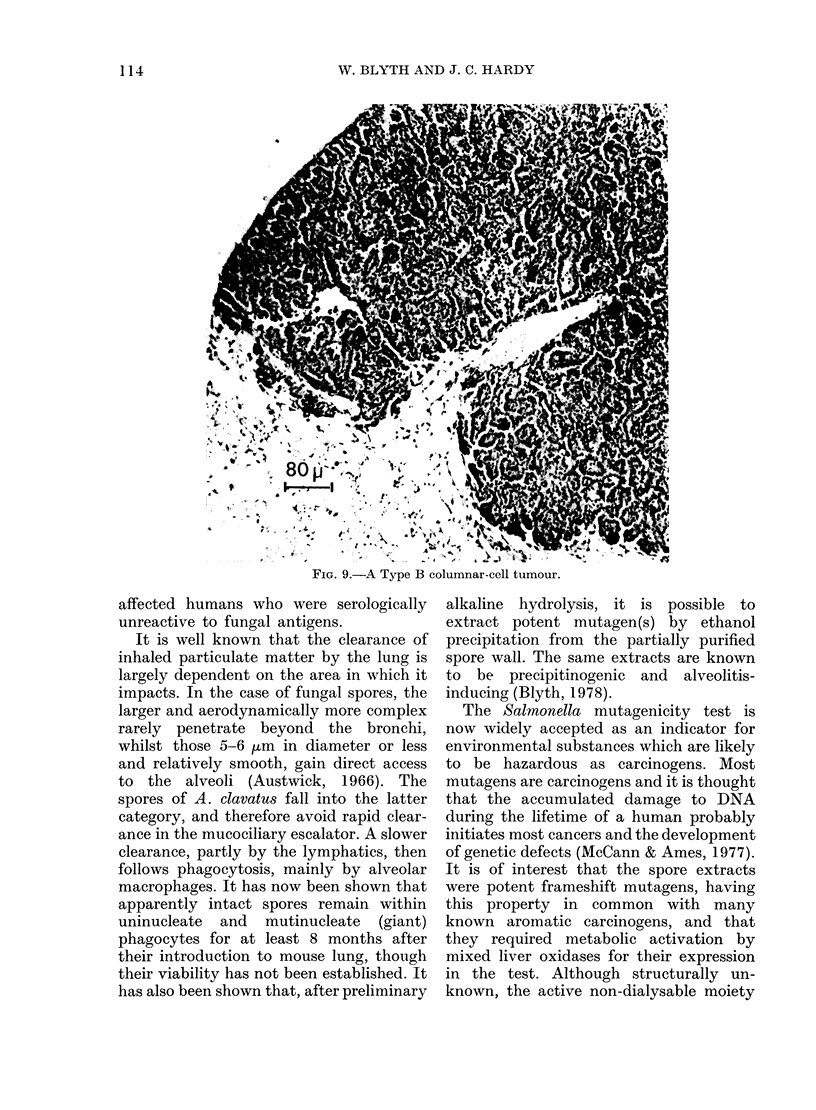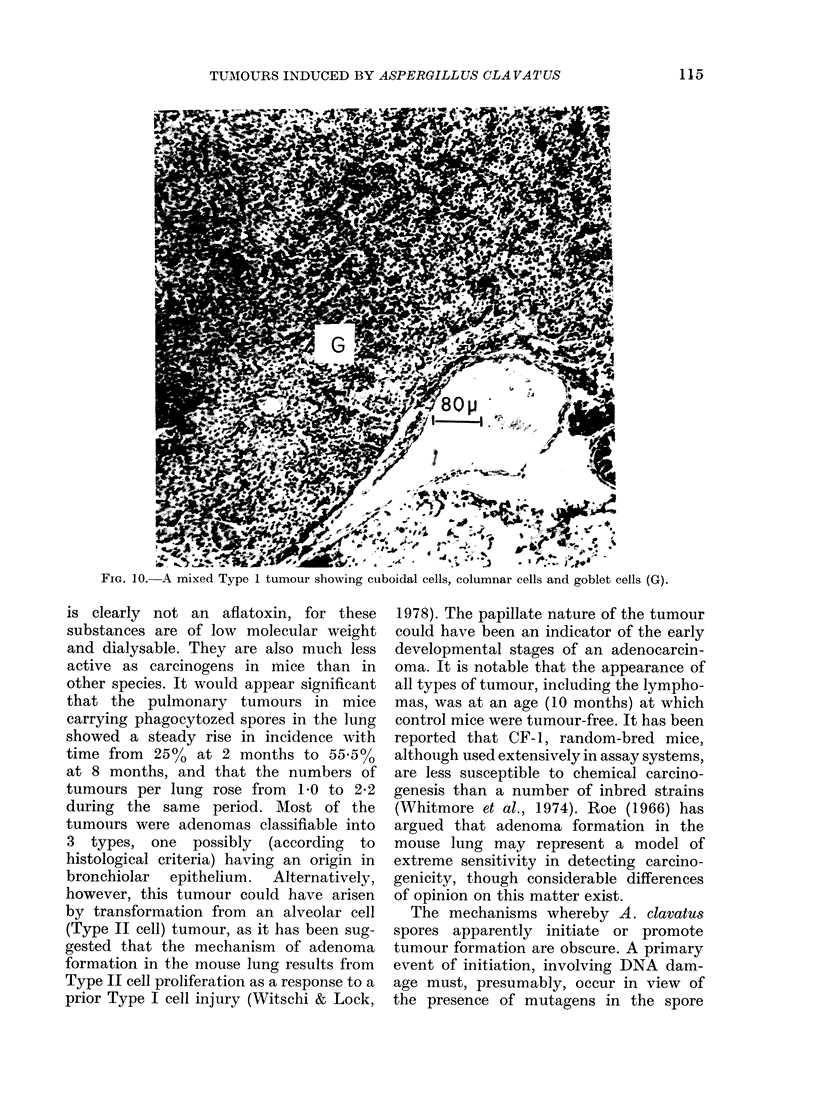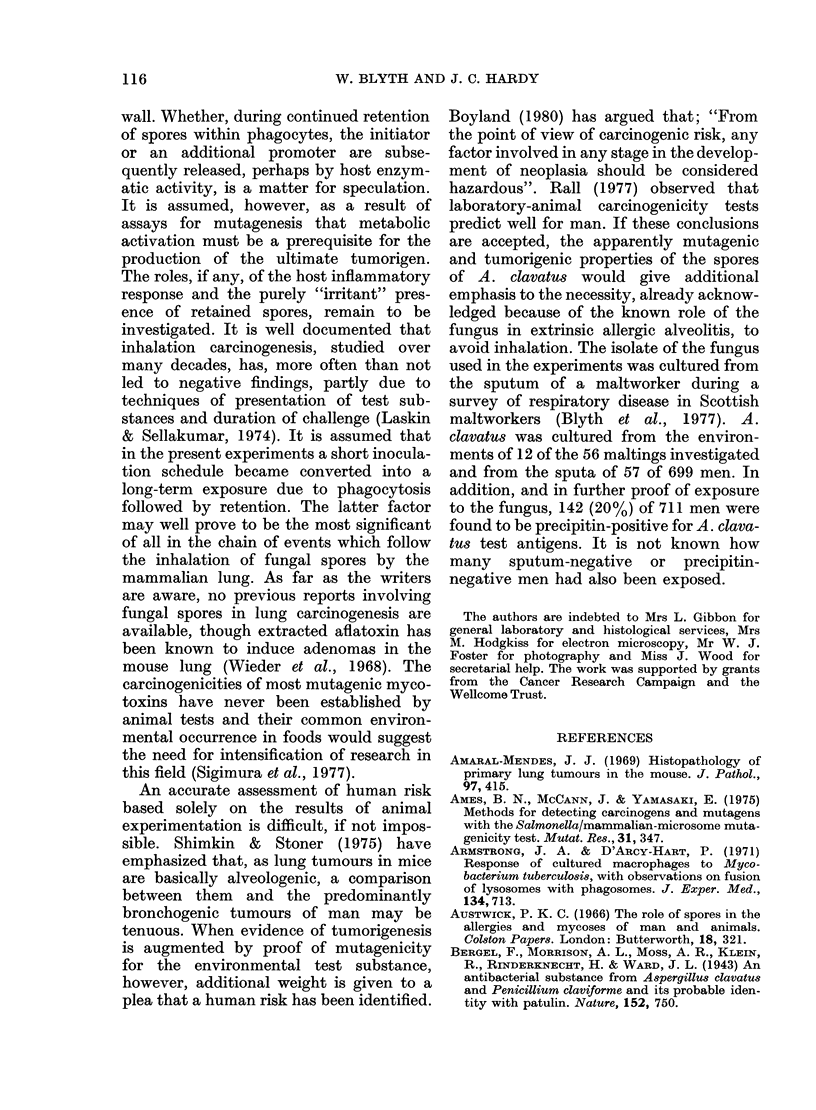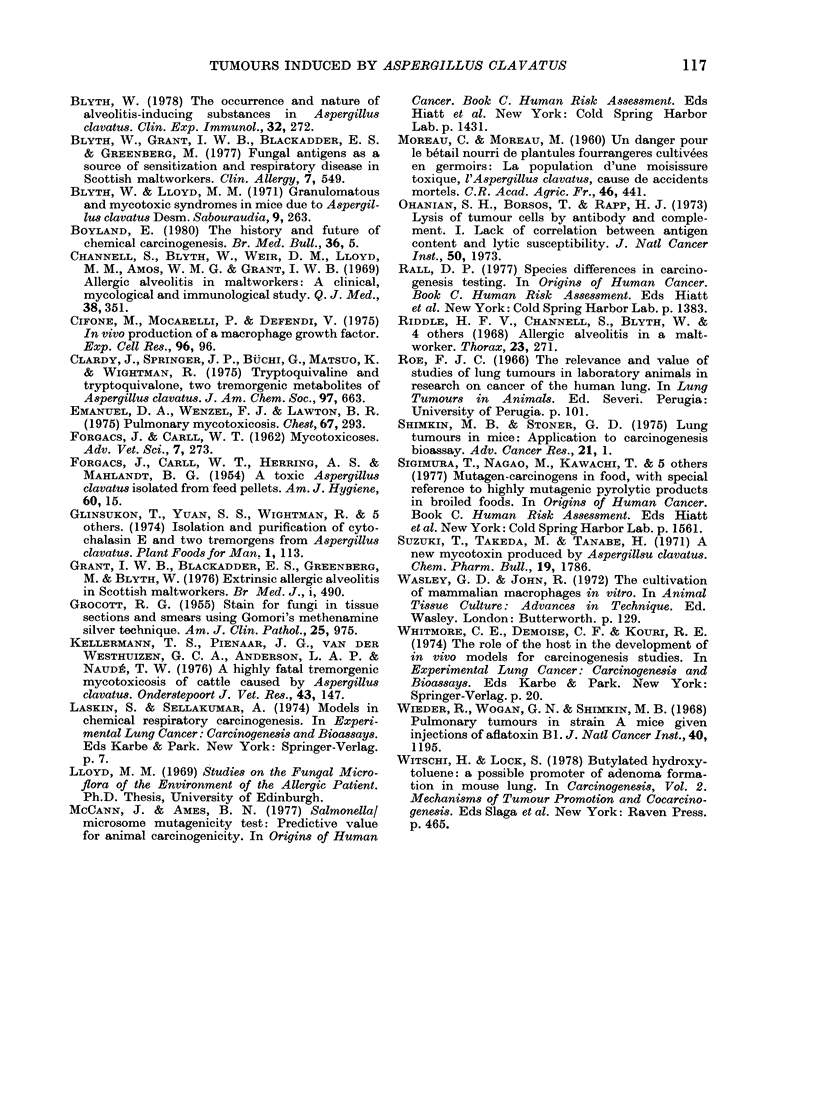# Mutagenic and tumourigenic properties of the spores of Aspergillus clavatus.

**DOI:** 10.1038/bjc.1982.13

**Published:** 1982-01

**Authors:** W. Blyth, J. C. Hardy

## Abstract

**Images:**


*Br. J. Cancer* (1982) **45**, 105

### MUTAGENIC AND TUMOURIGENIC PROPERTIES OF THE SPORES OF *ASPERGILLUS CLAVATUS*

W. BLYTH AND J. C. HARDY

*From the Experimental Mycoses Unit, Department of Botany, University of Edinburgh, King's Buildings, Edinburgh EH9 3JH*

Received 3 September 1981 Accepted 17 September 1981

**Summary.—Spore walls of a sputum-derived isolate of *Aspergillus clavatus* yielded mutagen(s) when their extracts were fractionally precipitated with ethanol following alkaline hydrolysis. After spores were given by nasal inoculation to 6–8-week-old CF-1 mice, light and electron microscopy of lung sections showed that they had been readily phagocytozed by the polymorphonuclear leucocytes and alveolar macrophages mobilized during early allergic alveolitis in immunized mice. The formation of phagosomes was followed in thioglycollate-stimulated peritoneal macrophages grown *in vitro*. Unimmunised mice showed a comparable lung reaction, attributed to pulmonary mycotoxicosis, and revealed a rising incidence of lung tumours, from 25% at 2 months from inoculation, to 27·3% at 6 and to 55·5% at 8. Mean numbers of tumours per lung rose from 1·0 to 2·2. Total tumours, including lymphomas, reached a final incidence of 77·7% at 8 months, when control animals were tumour-free. Tumour development correlated with the retention of apparently intact spores within giant cells probably derived from aggregates of alveolar macrophages. The implications of these findings in the light of the known history of human exposure to such spores is discussed.**

*Aspergillus clavatus* is an allergenic and mycotoxic fungus, which, when inhaled as air-borne conidia (spores) released from contaminated barley malt is known to cause extrinsic allergic alveolitis in malt-workers (Riddle *et al.*, 1968; Channell *et al.*, 1969; Grant *et al.*, 1976; Blyth *et al.*, 1977). The disease can be induced experimentally in non-sensitized, sensitized precipitin-negative and sensitized precipitin-positive mice (Blyth, 1978). It is common practice to incorporate a number of by-products arising during the course of the malting process into cattle fodders. Components such as grist and flour from heavily contaminated maltings have been known to yield high plate counts of the fungus in culture (Lloyd, 1969). Forgacs *et al.* (1954) and Forgacs & Carll (1962) attributed haemorrhagic syndrome in poultry and hyperkeratosis in calves to ingestion of the fungus, and Moreau & Moreau (1960) recorded instances of inco-ordination and hepatic degeneration in cattle after a feed of affected cereal seedlings. In the Transvaal, a fatal tremorgenic disease in cattle involving the deaths of 130 out of a herd of 330 followed ingestion of a ration of contaminated sorghum beer residue (Kellermann *et al.*, 1976). The almost invariably fatal Reyes syndrome in young children in the Far East has been reported to follow the ingestion of mouldy glutinous rice, and *A. clavatus* is probably the main organism concerned in the synthesis of the causal mycotoxins (Glinsukon *et al.*, 1974). The mycotoxins identified from cultures of the fungus grown under a variety of different environmental conditions and on different substrates, include patulin (Bergel *et al.*, 1943) escladiol (Suzuki *et al.*, 1971) cytochalasin E (Glinsukon *et al.*, 1974) and 2 tremorgens, tryptoquivaline and tryptoquivalone (Glinsukon *et al.*, 1974; Clardy *et al.*, 1975). Intraperitoneal injections of spore suspensions or killed mycelium have been shown to provoke hyperplastic liver nodules in mice, and ingestion of inoculated barley to induce hyperkeratosis (Blyth & Lloyd, 1971).

The following paper outlines the results of further investigations on experimental murine respiratory disease, provoked by the observation that, during early investigations of maltworkers, volunteers were found to remain sputum-positive for the fungus for at least 1 month after a single exposure to the air of a maltings whilst contaminated malt, releasing spores of the fungus, was being turned (Channell *et al.*, 1969). The work was designed to investigate the long-term effects of intranasal inoculations on the mouse lung.

### MATERIALS AND METHODS

*Sources of* Aspergillus clavatus.—During a survey of respiratory disease in Scottish maltworkers (Blyth *et al.*, 1977) cultures were obtained from the environments of maltings and others from the sputa of maltworkers. Amongst those retained for further study, one, designated isolate 301, was sputum-derived, and on culture in liquid media synthesized moeities extremely toxic to bacterial and mammalian cells. This isolate was used in the experiments reported here.

*Fungal culture.*—Cultures were grown in 1l flasks in aliquots of 250ml of Czapek Dox liquid medium (Oxoid, Ltd., Basingstoke, Hampshire) supplemented with 5g/l of casein hydrolysate (Sigma Chemical Co. Ltd, St Louis, Mo, U.S.A.). They were grown for 28 days in an orbital incubator in darkness at 26°C. Culture filtrate (CF) and mycelium were separated by centrifugation at 3000 *g* for 20 min. CF was concentrated ×40 by ultrafiltration using DP06 and UM2 filters in an Amicon-stirred cell, Model 202 (Amicon Ltd, Lexington, Mass, U.S.A.). Mycelium, mixed with a little sterile phosphate-buffered saline (PBS), was fragmented in a ball mill for 1 h, pelleted by centrifugation and passed through a Hughes pressure cell. The product was shaken in PBS for 5 days and centrifuged at 10,000 *g* for 20 min to remove cell fragments. The supernatant was concentrated by ultrafiltration to give an intracellular mycelial extract (M). Equal portions of CF and M were mixed for use as antigen in immunization schedules.

Some cultures were also grown in 1l Roux bottles on Czapek Dox/Casein hydrolysate medium supplemented with 2% Davis agar and were kept in darkness at 26°C for 10–14 days. Spores were harvested into sterile PBS by rubbing with a smoothed glass rod and the saline suspension was filtered through sterile gauze to remove fragments of mycelium and conidiophores. The spores were pelleted by centrifugation and washed ×3 by centrifugation in fresh aliquots of sterile PBS.

*Chemical extraction of spores.*—Aliquots of 3 ml of concentrated spore suspension were placed in 100 × 16 mm round-bottomed culture tubes (Sterilin, Teddington, Middx) to each of which was added a sample of acid-washed 40-mesh glass beads. The tubes were capped and run at full speed on a Griffin flask shaker (Griffin & George, Middx) for 30 min, ensuring cell breakage of at least 95%. Intracellular spore extract was separated from cell debris and beads by centrifugation and concentrated 5-fold by ultrafiltration with a UM2 filter. Crude cell walls were separated from glass beads by shaking in sterile PBS and decantation. They were pelleted and washed by centrifugation and sequentially extracted according to the method described by Blyth (1978). The method involved delipidation using chloroform:methanol, 1:1(v/v), digestion with trypsin and DNAse and hydrolysis in 3% NaOH at 100°C for 6 h. After centrifugation at this stage, the supernatant hydrolysate was fractionally precipitated with ethanol in the ranges 0–40, 40–60 and finally 60–80%. The precipitates were collected by centrifugation at 10,000 *g*, each being resuspended in distilled water and extensively dialysed over a period of 3 days. The hydrolysed cell walls were partially solubilized in 72% sulphuric acid for 48 h at 25°C. After centrifugation, the supernatant was neutralized and dialysed against distilled water.

*Macrophage culture and phagocytosis.*—Populations of stimulated macrophages were induced to aggregate in the peritoneal cavities of CF-1 mice by single i.p. injections of 1·5 ml fluid thioglycollate medium (Difco Laboratories, Detroit, Michigan, U.S.A.) The stimulated cells were withdrawn in sterile PBS after an interval of 4 days, according to the method of Wasley & John (1972). The cells were pelleted by centrifugation at 500 *g*

and aliquots of dense suspensions in fresh sterile PBS were introduced to 1ml volumes of Medium 199 (Flow Laboratories, Irvine, Scotland) in sterile, flat-based culture tubes (NUNC, Denmark) containing fragments of alcohol-sterilized mica. After 2–3 h, when macrophages had become firmly attached, residual suspended cells were removed by repeated washing in medium. Growth medium was then added in the form of Earle's Minimal Essential Medium (MEM) containing 2mM glutamine, 1% non-essential amino acids, 10% foetal bovine serum, and, on the recommendation of Cifone *et al.* (1975) 10% cell-free ascitic tumour fluid. Cells were held in culture for up to 7 days before use in phagocytosis experiments. Drops of fungal-spore suspension in M199 were added to established macrophage monolayers, and phagocytosis was allowed to proceed for 1–6 h. Selected material was taken for light and electron microscopy.

*Cytotoxicity tests.*—Evidence for the cytotoxicity of fungal extracts was sought firstly by estimating their effects on Ehrlich ascites tumour cells obtained from CF1 mice in which they were passaged at 7-day intervals. Cell suspensions in M199 were seeded into 96-well, culture-grade microtitre plates (Sterilin, Teddington, Middx.). The cells were cultured in a 5% $CO_2$ atmosphere in a humidified incubator at 37°C, using a medium containing MEM supplemented with 2mM glutamine, 1% non-essential amino acids and 10% foetal bovine serum. After tumour cells had been established for 24 h, the medium in the culture wells was aspirated and replaced for washing, by M199. The test materials, as 10 μl samples, were then added to wells in fresh M199 for 1 h. After washing, complete medium was then added and cytotoxicity estimated 24 h later. Uniform incubation was used throughout the test period. In a second test, human HEp2 cells were established in 1ml volumes of complete culture medium as above, but buffered with 20mM HEPES and held in air at 37°C. The schedule of testing was as before. In all cases trypan-blue exclusion was taken as the measure of cell-membrane integrity (Ohanian *et al.*, 1973).

*Mutagenesis.*—Spot tests and plate incorporation assays were conducted using the his⁻ strains, TA98 and TA100, of *Salmonella typhimurium* kindly supplied by Dr Bruce Ames (Department of Biochemistry, University of California, Berkley, California). Assays were performed in the presence or absence of mixed liver oxidases, provoked in CF1 mice by i.p. injection of the polychlorinated biphenyl mixture Aroclor (Monsanto Co., St Louis, Mo, U.S.A.). Aseptically excised livers were homogenized and centrifuged at 9000 *g* to provide microsomal preparations (S-9 fractions) which were mixed with co-factors to give an S-9 mix (Ames *et al.*, 1975). In addition to *A. clavatus* test extracts, Aflatoxin B1 and N-methyl-N′-nitro-N-nitrosoguanidine were used as controls (Sigma, St Louis, Mo, U.S.A.).

*Mice.*—Six to 8-week-old CF-1 mice were supplied by the Centre for Laboratory Animals, The Bush, Milton Bridge, Penicuik.

*Immunization and fungal inoculation.*—A short-term preliminary experiment, set up to establish the degree of murine allergic response to the spores of isolate 301, involved a group of 10 male and 10 female mice. Five males and 5 females were immunized with an i.p. injection of 0·1 ml fungal antigen, as described above, emulsified with an equal volume of Freund's Complete Adjuvant. An s.c. injection of the same mixture was given after 60 days. After a further 7 days, serum from blood samples obtained from the retro-orbital plexus was tested for the presence of precipitins by routine immunoelectrophoresis. The remaining unimmunized mice were also tested.

Three drops of spore suspension in sterile PBS were given by nasal inoculation on each of 4 successive days to mice lightly anaesthetized by ether. Each animal received a total dosage of $5–6 \times 10^5$ spores. All animals were killed 24 h after the last inoculation.

A further 28 female animals were given an identical schedule of inoculations for long-term assessments of pulmonary disease. Eight animals were killed at 2 months, 11 at 6 months and 9 at 8 months. Twenty control, untreated mice were examined for tumours at 9–10 months of age.

*Histology.*—Lungs were excised after preliminary irrigation with phosphate-buffered 10% formaldehyde (pH 7·0) given by intratracheal instillation. After dehydration and embedding, serial sections were cut and routinely stained with haematoxylin and eosin. To allow visualization of fungal elements, some sections were stained by the Gomori–Grocott methenamine-silver nitrate method (Grocott, 1955) and others by the periodic acid-Schiff technique.

*Electron microscopy.*—Sections were made of alveolar cells harvested in PBS by intratracheal lavage from the lungs of mice used in the short-term experiment. The cells were pelleted by low-speed centrifugation and fixed in 2·5% glutaraldehyde in 0·1M sodium cacodylate buffer, pH 7·4. Further processing was essentially as described by Armstrong & D'Arcy-Hart (1971). Macrophages grown *in vitro* on sheets of mica were fixed in 3% glutaraldehyde in phosphate buffer (pH 7·2) and the sheets were trimmed to convenient size before embedding in Araldite. Tissues from the lungs of mice in the acute phase of *A. clavatus* alveolitis were processed similarly to macrophage monolayers. Sections cut on an LKB Ultrotome (LKB Produkter AB, Sweden) were stained with lead citrate or lead citrate and uranyl acetate, prior to examination on an AEI electron microscope (Kratos, AEI Scientific Instruments, Manchester).

### RESULTS

### In vitro *phagocytosis and animal inoculations*

Twenty-four hours after the schedule of nasal inoculations with fungal spores, the lungs of both immunized and unimmunized mice showed severe perivasculitis and peribronchiolitis. In addition to widespread infiltration of alveolar septa by inflammatory cells, numerous and sometimes extensive areas of alveolar filling were randomly distributed in the parenchyma of most lobes. The histology accorded with that of Grade 5 *A. clavatus*-induced allergic alveolitis as described by Blyth (1978) and was clearly the most severe yet seen in unimmunized mice, in which isolate 301 had not previously been used. No differences in severity were observed between males and females.

Most inflammatory exudates in immunized animals were predominantly of macrophages, polymorphonuclear cells being more abundant in the lungs of the unimmunized. From the electron microscopy of cells obtained from lung washings (Fig. 1) from thin sections of lung tissue (Fig. 2) and from peritoneal cells grown *in vitro* (Figs 3 & 4) phagocytosis by both polymorphonuclear leucocytes and macrophages was readily identifiable. The spores were contained in phagosomes delimited by unit membranes from the surrounding cell contents, which showed good resolution of most organelles: nucleus, mitochondria, Golgi apparatus and endoplasmic reticulum. Most phagosome membranes were closely appressed to the engulfed spores, but rare cases of "loose" phagosomes were seen, in which a wide electron-lucent area lay between membrane and spore wall (Fig. 1). Phagocytozed spores, observed at high magnification, were seen to have a 3–4-layered wall, the outermost layer showing as a thin, electron-dense covering (Fig. 4). The organelles of the spores were variably preserved. In some phagocytes, vacuolar bodies showing complexes of unidentified lamellate material were clearly visualized. Lung cultures taken at this stage were usually positive for the fungus.

Information from all the remaining animals of the experiment is summarized in Table 1. Two months after inoculation, focal inflammatory infiltrates had largely resolved. The few remaining were mostly perivascular or peribronchiolar and predominantly lymphocytic. In these cell aggregates, alveolar macrophages were occasionally seen as single, clearly separate cells containing phagocytozed and apparently intact spores. A characteristic feature, however, was the occurrence of phagocytic multinucleate cells (giant cells) showing no delineation of internal cell membranes in sections stained with haematoxylin and eosin. When stained by the methenamine-silver method, giant cells were also seen to contain intact spores (Fig. 5). The origin of the cells would appear to have been by fusion of a group of individual macrophages. Lung cultures from such material were negative. In 2 of the 8 animals killed at this stage, however, small tumours, sub-pleural in position and too small to be seen at postmortem examination were seen in lung sections. Nine of the 11 animals killed at 6 months had randomly distributed phagocytic giant cells, and 3 retained residual

FIG. 1.—Section of a cell aggregate obtained by tracheo-bronchial lavage, showing a polymorphonuclear leucocyte containing a fungal spore within a "loose" phagosome.

TABLE I.—*Tumour incidence in CF-1 mice given spores of* Aspergillus clavatus *by nasal inoculation*

| No. mice inoculated | Months after inoculation | No. mice with giant cells containing spores | No. mice with tumours: Lymphocytic | Lung | Nos. lung tumours per mouse |
|---|---|---|---|---|---|
| 8 | 2 | 6 | 0 | 2 (25)* | 1·0 |
| 11 | 6 | 9 | 0 | 3 (27·3) | 1·0 |
| 9 | 8 | 6 | 2 (22·2) | 5 (55·5) | 2·2 |

* Figures in parentheses are percentages.

areas of mild alveolitis. Lymphocytic perivasculitis was a common feature involving all animals. Double tumours were identified in 1 mouse (Fig. 6) and single tumours in 2 more (Fig. 7). Small areas of bronchiolar metaplasia were seen in 2 animals. The remaining 9 animals in the experiment were killed at 8 months. Two had developed lymphoma, each showing enlarged thymus and spleen. Five other animals had developed lung tumours; 3 tumours per lung were seen in 2 of the mice, and 2 tumours per lung in 2 more, and a single tumour in the remaining animal. Cultures were negative.

*Lung tumours*

Based upon the classification outlined by Amaral-Mendes (1969) 3 distinct types of pulmonary tumour were identified. Seven of the 10 affected mice showed Type A, cuboidal-cell tumours derived from alveo-

FIG. 2.—Fungal spores in "tight" phagosomes within macrophage cytoplasm in a lung section.

lar epithelium and developing by intra-alveolar proliferation (Fig. 8). The peripheries of the tumours apparently encroached progressively on surrounding parenchymal tissue by continued metaplasia of alveolar epithelium. The tumour cells characteristically had a nucleus-cytoplasm ratio $<1$, had strongly acidophilic cytoplasm, were PAS-negative and showed few mitotic figures. Type B, columnar-cell tumours, probably of bronchiolar epithelial origin were seen in 1 animal and showed more invasive, papillate growth from (Fig. 9). Cells were basophilic with large nuclei, many in stages of division, with a nucleus/cytoplasm ratio $>1$. They were PAS-positive. The remaining animal of the series had a mixed-cell tumour of Type 1, containing elements of Types A and B and with islands of goblet cells (Fig. 10).

No tumours were found in the 20 control mice killed when 9–10 months old.

*Cytotoxicity tests*

Ehrlich ascites and HEp 2 cells showed uniformity of reaction during the tests. Intracellular spore extracts and the products of acid hydrolysis of spore walls, were consistently non-toxic, even though the latter are known to be tremorgenic (Blyth, 1978; Blyth & Lloyd, 1971). Redissolved and dialysed ethanol precipitates from alkaline hydrolysis were highly toxic, the 0–40% and 40–60% extracts being more uniformly so during extractions involving various different batches of spores. All cells were usually dead, and many had detached, by the end of the test period.

*Mutagenicity tests*

No evidence of mutagenicity was obtained using either intracellular spore extracts or the products of acid hydrolysis of spore walls. All 3 ranges of ethanol precipitation following the alkaline hydro-

FIG. 3.—Early phagocytosis of a spore by plate-like extensions, here seen in section, at the surface of a thioglycollate-stimulated peritoneal macrophage.

FIG. 4.—Multi-layered wall (W) of a spore (SP) in a "tight" phagosome of a peritoneal macrophage.

FIG. 5.—An alveolar giant cell (G) containing 3 spores (SP) stained by the methenamine-silver method.

FIGS 6 and 7.—Double and single tumours in lungs from mice 6 months after fungal inoculation.

FIG. 8.—A Type A cuboidal-cell tumour.

lysis of the walls, however, showed mutagenic activity, significantly increasing mean numbers of revertants of strains TA98 and TA100 of *S. typhimurium*. As is shown in Table II, mutagenic expression required activation by liver microsomal (S-9) fractions.

TABLE II.—*Mean numbers of revertants of* S. typhimurium *strains TA*98 *and TA*100 *in the presence or absence of mouse liver microsomal extracts* (*S*-9) *following exposure to ethanol precipitates obtained from the products of alkaline hydrolysis of spore walls of* Aspergillus clavatus.

| | TA 98 | | TA 100 | |
|---|---|---|---|---|
| | S-9 mix | | S-9 mix | |
| | − | + | − | + |
| Spontaneous revertants (control) | 18 | | 96 | |
| Ethanol precipitates in the range of: | | | | |
| 0–40% | 16 | 329 | 26 | 346 |
| 40–60% | 18 | 306 | 30 | 268 |
| 60–80% | 16 | 331 | 26 | 285 |

### DISCUSSION

It has been shown that an isolate of of *A. clavatus* capable of synthesizing metabolites highly toxic to bacterial and mammalian cells will provoke an inflammatory response in the lungs of unimmunized mice. A similar response in immunized mice has been attributed to a mixed Type III (Arthus) and Type IV hypersensitivity reaction (Blyth, 1978). In the absence of an immunological basis for this response, Emanuel *et al.* (1975) have suggested that the pulmonary disease provoked by massive inhalation of fungal spores might be described as a pulmonary mycotoxicosis. The unsensitized animals examined 24 h after fungal inoculation showed an inflammatory reaction grossly similar to that described in

FIG. 9.—A Type B columnar-cell tumour.

affected humans who were serologically unreactive to fungal antigens.

It is well known that the clearance of inhaled particulate matter by the lung is largely dependent on the area in which it impacts. In the case of fungal spores, the larger and aerodynamically more complex rarely penetrate beyond the bronchi, whilst those 5–6 $\mu$m in diameter or less and relatively smooth, gain direct access to the alveoli (Austwick, 1966). The spores of *A. clavatus* fall into the latter category, and therefore avoid rapid clearance in the mucociliary escalator. A slower clearance, partly by the lymphatics, then follows phagocytosis, mainly by alveolar macrophages. It has now been shown that apparently intact spores remain within uninucleate and mutinucleate (giant) phagocytes for at least 8 months after their introduction to mouse lung, though their viability has not been established. It has also been shown that, after preliminary alkaline hydrolysis, it is possible to extract potent mutagen(s) by ethanol precipitation from the partially purified spore wall. The same extracts are known to be precipitinogenic and alveolitis-inducing (Blyth, 1978).

The *Salmonella* mutagenicity test is now widely accepted as an indicator for environmental substances which are likely to be hazardous as carcinogens. Most mutagens are carcinogens and it is thought that the accumulated damage to DNA during the lifetime of a human probably initiates most cancers and the development of genetic defects (McCann & Ames, 1977). It is of interest that the spore extracts were potent frameshift mutagens, having this property in common with many known aromatic carcinogens, and that they required metabolic activation by mixed liver oxidases for their expression in the test. Although structurally unknown, the active non-dialysable moiety

FIG. 10.—A mixed Type 1 tumour showing cuboidal cells, columnar cells and goblet cells (G).

is clearly not an aflatoxin, for these substances are of low molecular weight and dialysable. They are also much less active as carcinogens in mice than in other species. It would appear significant that the pulmonary tumours in mice carrying phagocytozed spores in the lung showed a steady rise in incidence with time from 25% at 2 months to 55·5% at 8 months, and that the numbers of tumours per lung rose from 1·0 to 2·2 during the same period. Most of the tumours were adenomas classifiable into 3 types, one possibly (according to histological criteria) having an origin in bronchiolar epithelium. Alternatively, however, this tumour could have arisen by transformation from an alveolar cell (Type II cell) tumour, as it has been suggested that the mechanism of adenoma formation in the mouse lung results from Type II cell proliferation as a response to a prior Type I cell injury (Witschi & Lock, 1978). The papillate nature of the tumour could have been an indicator of the early developmental stages of an adenocarcinoma. It is notable that the appearance of all types of tumour, including the lymphomas, was at an age (10 months) at which control mice were tumour-free. It has been reported that CF-1, random-bred mice, although used extensively in assay systems, are less susceptible to chemical carcinogenesis than a number of inbred strains (Whitmore *et al.*, 1974). Roe (1966) has argued that adenoma formation in the mouse lung may represent a model of extreme sensitivity in detecting carcinogenicity, though considerable differences of opinion on this matter exist.

The mechanisms whereby *A. clavatus* spores apparently initiate or promote tumour formation are obscure. A primary event of initiation, involving DNA damage must, presumably, occur in view of the presence of mutagens in the spore wall. Whether, during continued retention of spores within phagocytes, the initiator or an additional promoter are subsequently released, perhaps by host enzymatic activity, is a matter for speculation. It is assumed, however, as a result of assays for mutagenesis that metabolic activation must be a prerequisite for the production of the ultimate tumorigen. The roles, if any, of the host inflammatory response and the purely "irritant" presence of retained spores, remain to be investigated. It is well documented that inhalation carcinogenesis, studied over many decades, has, more often than not led to negative findings, partly due to techniques of presentation of test substances and duration of challenge (Laskin & Sellakumar, 1974). It is assumed that in the present experiments a short inoculation schedule became converted into a long-term exposure due to phagocytosis followed by retention. The latter factor may well prove to be the most significant of all in the chain of events which follow the inhalation of fungal spores by the mammalian lung. As far as the writers are aware, no previous reports involving fungal spores in lung carcinogenesis are available, though extracted aflatoxin has been known to induce adenomas in the mouse lung (Wieder *et al.*, 1968). The carcinogenicities of most mutagenic mycotoxins have never been established by animal tests and their common environmental occurrence in foods would suggest the need for intensification of research in this field (Sigimura *et al.*, 1977).

An accurate assessment of human risk based solely on the results of animal experimentation is difficult, if not impossible. Shimkin & Stoner (1975) have emphasized that, as lung tumours in mice are basically alveologenic, a comparison between them and the predominantly bronchogenic tumours of man may be tenuous. When evidence of tumorigenesis is augmented by proof of mutagenicity for the environmental test substance, however, additional weight is given to a plea that a human risk has been identified. Boyland (1980) has argued that; "From the point of view of carcinogenic risk, any factor involved in any stage in the development of neoplasia should be considered hazardous". Rall (1977) observed that laboratory-animal carcinogenicity tests predict well for man. If these conclusions are accepted, the apparently mutagenic and tumorigenic properties of the spores of *A. clavatus* would give additional emphasis to the necessity, already acknowledged because of the known role of the fungus in extrinsic allergic alveolitis, to avoid inhalation. The isolate of the fungus used in the experiments was cultured from the sputum of a maltworker during a survey of respiratory disease in Scottish maltworkers (Blyth *et al.*, 1977). *A. clavatus* was cultured from the environments of 12 of the 56 maltings investigated and from the sputa of 57 of 699 men. In addition, and in further proof of exposure to the fungus, 142 (20%) of 711 men were found to be precipitin-positive for *A. clavatus* test antigens. It is not known how many sputum-negative or precipitin-negative men had also been exposed.

The authors are indebted to Mrs L. Gibbon for general laboratory and histological services, Mrs M. Hodgkiss for electron microscopy, Mr W. J. Foster for photography and Miss J. Wood for secretarial help. The work was supported by grants from the Cancer Research Campaign and the Wellcome Trust.

### REFERENCES

Amaral-Mendes, J. J. (1969) Histopathology of primary lung tumours in the mouse. *J. Pathol.*, **97**, 415.

Ames, B. N., McCann, J. & Yamasaki, E. (1975) Methods for detecting carcinogens and mutagens with the *Salmonella*/mammalian-microsome mutagenicity test. *Mutat. Res.*, **31**, 347.

Armstrong, J. A. & D'Arcy-Hart, P. (1971) Response of cultured macrophages to *Mycobacterium tuberculosis*, with observations on fusion of lysosomes with phagosomes. *J. Exper. Med.*, **134**, 713.

Austwick, P. K. C. (1966) The role of spores in the allergies and mycoses of man and animals. *Colston Papers*. London: Butterworth, **18**, 321.

Bergel, F., Morrison, A. L., Moss, A. R., Klein, R., Rinderknecht, H. & Ward, J. L. (1943) An antibacterial substance from *Aspergillus clavatus* and *Penicillium claviforme* and its probable identity with patulin. *Nature*, **152**, 750.

BLYTH, W. (1978) The occurrence and nature of alveolitis-inducing substances in *Aspergillus clavatus*. *Clin. Exp. Immunol.*, **32**, 272.

BLYTH, W., GRANT, I. W. B., BLACKADDER, E. S. & GREENBERG, M. (1977) Fungal antigens as a source of sensitization and respiratory disease in Scottish maltworkers. *Clin. Allergy*, **7**, 549.

BLYTH, W. & LLOYD, M. M. (1971) Granulomatous and mycotoxic syndromes in mice due to *Aspergillus clavatus* Desm. *Sabouraudia*, **9**, 263.

BOYLAND, E. (1980) The history and future of chemical carcinogenesis. *Br. Med. Bull.*, **36**, 5.

CHANNELL, S., BLYTH, W., WEIR, D. M., LLOYD, M. M., AMOS, W. M. G. & GRANT, I. W. B. (1969) Allergic alveolitis in maltworkers: A clinical, mycological and immunological study. *Q. J. Med.*, **38**, 351.

CIFONE, M., MOCARELLI, P. & DEFENDI, V. (1975) *In vivo* production of a macrophage growth factor. *Exp. Cell Res.*, **96**, 96.

CLARDY, J., SPRINGER, J. P., BÜCHI, G., MATSUO, K. & WIGHTMAN, R. (1975) Tryptoquivaline and tryptoquivalone, two tremorgenic metabolites of *Aspergillus clavatus*. *J. Am. Chem. Soc.*, **97**, 663.

EMANUEL, D. A., WENZEL, F. J. & LAWTON, B. R. (1975) Pulmonary mycotoxicosis. *Chest*, **67**, 293.

FORGACS, J. & CARLL, W. T. (1962) Mycotoxicoses. *Adv. Vet. Sci.*, **7**, 273.

FORGACS, J., CARLL, W. T., HERRING, A. S. & MAHLANDT, B. G. (1954) A toxic *Aspergillus clavatus* isolated from feed pellets. *Am. J. Hygiene*, **60**, 15.

GLINSUKON, T., YUAN, S. S., WIGHTMAN, R. & 5 others. (1974) Isolation and purification of cytochalasin E and two tremorgens from *Aspergillus clavatus*. *Plant Foods for Man*, **1**, 113.

GRANT, I. W. B., BLACKADDER, E. S., GREENBERG, M. & BLYTH, W. (1976) Extrinsic allergic alveolitis in Scottish maltworkers. *Br Med. J.*, i, 490.

GROCOTT, R. G. (1955) Stain for fungi in tissue sections and smears using Gomori's methenamine silver technique. *Am. J. Clin. Pathol.*, **25**, 975.

KELLERMANN, T. S., PIENAAR, J. G., VAN DER WESTHUIZEN, G. C. A., ANDERSON, L. A. P. & NAUDÉ, T. W. (1976) A highly fatal tremorgenic mycotoxicosis of cattle caused by *Aspergillus clavatus*. *Onderstepoort J. Vet. Res.*, **43**, 147.

LASKIN, S. & SELLAKUMAR, A. (1974) Models in chemical respiratory carcinogenesis. In *Experimental Lung Cancer: Carcinogenesis and Bioassays*. Eds Karbe & Park. New York: Springer-Verlag. p. 7.

LLOYD, M. M. (1969) *Studies on the Fungal Microflora of the Environment of the Allergic Patient*. Ph.D. Thesis, University of Edinburgh.

MCCANN, J. & AMES, B. N. (1977) *Salmonella/* microsome mutagenicity test: Predictive value for animal carcinogenicity. In *Origins of Human Cancer. Book C. Human Risk Assessment*. Eds Hiatt *et al.* New York: Cold Spring Harbor Lab. p. 1431.

MOREAU, C. & MOREAU, M. (1960) Un danger pour le bétail nourri de plantules fourrangeres cultivées en germoirs: La population d'une moisissure toxique, *l'Aspergillus clavatus*, cause de accidents mortels. *C.R. Acad. Agric. Fr.*, **46**, 441.

OHANIAN, S. H., BORSOS, T. & RAPP, H. J. (1973) Lysis of tumour cells by antibody and complement. I. Lack of correlation between antigen content and lytic susceptibility. *J. Natl Cancer Inst.*, **50**, 1973.

RALL, D. P. (1977) Species differences in carcinogenesis testing. In *Origins of Human Cancer. Book C. Human Risk Assessment*. Eds Hiatt *et al.* New York: Cold Spring Harbor Lab. p. 1383.

RIDDLE, H. F. V., CHANNELL, S., BLYTH, W. & 4 others (1968) Allergic alveolitis in a maltworker. *Thorax*, **23**, 271.

ROE, F. J. C. (1966) The relevance and value of studies of lung tumours in laboratory animals in research on cancer of the human lung. In *Lung Tumours in Animals*. Ed. Severi. Perugia: University of Perugia. p. 101.

SHIMKIN, M. B. & STONER, G. D. (1975) Lung tumours in mice: Application to carcinogenesis bioassay. *Adv. Cancer Res.*, **21**, 1.

SIGIMURA, T., NAGAO, M., KAWACHI, T. & 5 others (1977) Mutagen-carcinogens in food, with special reference to highly mutagenic pyrolytic products in broiled foods. In *Origins of Human Cancer*. Book C. *Human Risk Assessment*. Eds Hiatt *et al.* New York: Cold Spring Harbor Lab. p. 1561.

SUZUKI, T., TAKEDA, M. & TANABE, H. (1971) A new mycotoxin produced by *Aspergillsu clavatus*. *Chem. Pharm. Bull.*, **19**, 1786.

WASLEY, G. D. & JOHN, R. (1972) The cultivation of mammalian macrophages *in vitro*. In *Animal Tissue Culture: Advances in Technique*. Ed. Wasley. London: Butterworth. p. 129.

WHITMORE, C. E., DEMOISE, C. F. & KOURI, R. E. (1974) The role of the host in the development of *in vivo* models for carcinogenesis studies. In *Experimental Lung Cancer: Carcinogenesis and Bioassays*. Eds Karbe & Park. New York: Springer-Verlag. p. 20.

WIEDER, R., WOGAN, G. N. & SHIMKIN, M. B. (1968) Pulmonary tumours in strain A mice given injections of aflatoxin B1. *J. Natl Cancer Inst.*, **40**, 1195.

WITSCHI, H. & LOCK, S. (1978) Butylated hydroxytoluene: a possible promoter of adenoma formation in mouse lung. In *Carcinogenesis, Vol. 2. Mechanisms of Tumour Promotion and Cocarcinogenesis*. Eds Slaga *et al.* New York: Raven Press. p. 465.